# Orthopedic surgery changes aortic valve composition in *ApoE−/−* mice

**DOI:** 10.1016/j.athplu.2026.100606

**Published:** 2026-09-02

**Authors:** Amber van Broekhoven, Wessel W. Fuijkschot, Ilse P.A. Zethof, Wessel N. van Wieringen, Yvo M. Smulders, Alexander B.A. Vonk, Paul A.J. Krijnen, Hans W.M. Niessen

**Affiliations:** a Department of Pathology, Amsterdam UMC, Vrije Universiteit, Amsterdam, the Netherlands; b Amsterdam Cardiovascular Sciences, Amsterdam, the Netherlands; c Department of Cardiac Surgery, Amsterdam UMC, Vrije Universiteit, Amsterdam, the Netherlands; d Department of Cardiology, Radboud University Medical Center, Nijmegen, the Netherlands; e Department of Epidemiology and Biostatistics, Amsterdam UMC, Vrije Universiteit, Amsterdam, the Netherlands; f Department of Mathematics, VU University, Amsterdam, the Netherlands; g Department of Internal Medicine, Amsterdam UMC, Vrije Universiteit, Amsterdam, the Netherlands

**Keywords:** Aortic stenosis, Atherosclerosis, Systemic inflammation, Non-cardiac surgery, Orthopedic surgery

## Abstract

**Background:**

In atherosclerotic cardiovascular disease (ASCVD), acute systemic inflammatory insults such as infections or major surgery are known to trigger cardiovascular events. However, it remains unclear whether such systemic triggers also modulate local inflammatory and degenerative processes within the aortic valve (AV). We therefore investigated the effect of orthopedic surgery on AV remodeling in an atherosclerosis-prone setting.

**Methods:**

ApoE−/− mice subjected to orthopedic surgery, ApoE−/− controls, and non-atherosclerotic controls were analyzed at 5 and 15 days post-intervention. AVs were assessed for valve thickness, fibrosis, glycosaminoglycans (GAGs), lipid accumulation, calcification, iron deposition, and inflammatory cell infiltration.

**Results:**

Atherosclerotic mice exhibited degenerative AV changes compared to non-atherosclerotic controls. Orthopedic surgery did not affect valve thickness or inflammatory cell infiltration, but induced compositional changes, including increased GAG content at day 5 (56.7 vs. 27.3; p = 0.016), a a higher fibrotic area at day 15 that did not reach statistical significance (41.3 vs. 24.9; p = 0.068), and reduced estimated lipid-rich area at day 5 (11.3 vs. 37.3; p = 0.008). No differences were observed in calcification or iron deposition.

**Conclusion:**

Orthopedic surgery induces early compositional remodeling of atherosclerotic aortic valves without increasing inflammatory cell infiltration or valve thickening. These findings suggest that orthopedic surgery induces compositional remodeling of the AV in an atherosclerotic setting. Although inflammatory cell infiltration was not increased at the examined time points, the underlying molecular mechanisms were not investigated. Therefore, systemic inflammatory mediators may contribute to these changes, but this remains to be established.

## Highlights

• Surgery induces early compositional remodeling of aortic valves.
• No increase in inflammatory cells or valve thickness was observed after surgery.
• Systemic mediators drive remodeling independent of cell infiltration.

## 1 Introduction

Calcific aortic valve disease (CAVD) is the most prevalent form of valvular heart disease in older adults in Western countries. It is characterized by sustained inflammation and progressive fibrocalcific changes of the aortic valve, ultimately leading to valve obstruction and impaired blood flow, clinically manifesting as aortic stenosis (AS) [1,2]. To date, no pharmacological therapies have demonstrated efficacy in halting or slowing the progression of AS, highlighting an urgent need for novel therapeutic approaches. CAVD was long considered a passive, age-related degenerative process. Increasingly evidence now support the view that CAVD represents an active, complex pathological process, consisting of two distinct phases. The initiation phase involves damage to the valve endothelial cell (VEC) layer, enabling the infiltration of circulating lipoproteins and immune cells. This is followed by the propagation phase, during which valve interstitial cells (VICs) are activated in response to stimulation and differentiate into myofibroblast- and osteoblast-like cells, leading to fibrosis and calcification [[3], [4], [5], [6], [7], [8]]. There is growing recognition that the early stages of CAVD resemble the pathophysiological mechanisms observed in atherosclerosis and its progression towards atherosclerotic cardiovascular disease (ASCVD) [3,[9], [10], [11], [12], [13]].

In ASCVD, observational epidemiological studies have shown that cardiovascular events can be triggered by a variety of common non-cardiovascular clinical conditions, particularly those linked to systemic inflammation. Notably, both systemic infections and major surgical interventions have been identified as key triggers [[14], [15], [16], [17]]. The acute rise in cardiovascular risk associated with these factors suggests they may influence atherosclerotic plaque stability, potentially by exacerbating or initiating local inflammatory processes within the arterial wall. Indeed, we previously demonstrated that systemic inflammation induced by major surgery caused an enlargement of the atherosclerotic plaque of the aortic root in an atherosclerotic mouse model, mainly due to an increase of the necrotic core [18].

Recent interest has also emerged in the possibility that these systemic insults may exacerbate or initiate local inflammatory processes within the AV as well. It has been observed that in patients with hip fractures requiring hip replacement, the presence of severe AS significantly increases the risk of mortality [[19], [20], [21], [22]]. Moreover, in patients with AS undergoing orthopedic surgery, the peak pressure gradient across the aortic valve is elevated, indicating a rapid clinical progression of the condition [23]. This concept, largely unexplored in the context of calcific aortic valve disease (CAVD), warrants further investigation. We now examined the impact of orthopedic surgery on aortic valve morphology and inflammation in an atherosclerotic mouse model.

## 2 Materials and methods

### 2.1 Murine surgery model

The present study represents a secondary, exploratory analysis of a previously conducted animal experiment. Whereas the original study focused on atherosclerotic plaque characteristics in the aortic root, the present analysis was designed to explore potential effects of orthopedic surgery on the aortic valve [18]. In short, 9-10 weeks old female *ApoE*^*−/−*^ mice (Charles River Laboratories, CalcoComo, Italy) were fed for 10 weeks on a high fat diet (0,15% cholesterol, 4022.83, AB diets, Woerden, the Netherlands) to induce atherosclerosis before allocation to the intervention groups; this design was based on the well-established susceptibility of ApoE−/− mice to rapid lesion development, with early foam-cell lesions arising within weeks and more advanced lesions developing thereafter, particularly under a Western-type diet [24,25]. Female mice were selected because young female ApoE−/− mice have been shown to develop robust atherosclerotic lesions, with lesion burden at least comparable to, and in some studies greater than, that of age-matched males [26]. Thereafter, the mice were divided into two groups: a non-surgical group (atherosclerotic control [AC] group, n = 13) and a group that underwent orthopedic surgery (atherosclerotic surgery [AS] group, n = 21). In the AC group, 7 mice were sacrificed at day 5 and 6 mice at day 15. In the AS group, 11 mice were sacrificed at day 5 and 10 mice at day 15. The AC group was injected intraperitoneally with NaCl_2_ 0.9% (5 mg/kg). The AS group underwent a midshaft femur osteotomy followed by realignment with a intramedullary K-wire. After a fasting period of 4 h, we sampled blood at several time points around orthopedic surgery/control (t = −1 day, 1 day, 5 days and 15 days). The mice were terminated 5 or 15 days after either surgery or saline injection by using a CO_2_ box (100% CO_2_). These time points were determined to analyze the effects of surgery on systemic inflammation in the acute (t = 5) and sub-acute phase (t = 15).

As a control, AVs from non-atherosclerotic *C5*7BL*/6* mice without surgery (C group, n = 6, 12 weeks old) were used. The mice were housed under standard conditions and exposed to a 12 h light/dark cycle and were fed with a normal non-fat diet. The procedures were approved by the Animal use and care Committee at the Amsterdam UMC, Vrije Universiteit, Amsterdam, the Netherlands.

### 2.2 Analysis of serum markers of inflammation, serum lipids, and body weight

To determine whether a systemic inflammatory response was induced within the ApoE−/− mice subjected to surgery, serum amyloid A (SAA) levels were measured by a commercially available ELISA (Life Technologies, Bleiswijk, the Netherlands) according to the manufacturers' protocol. SAA proteins are highly responsive acute-phase proteins mainly synthesized in the liver, with plasma concentrations increasing up to 1000-fold during inflammation. They play a central role in innate immunity by stimulating pro-inflammatory cytokines (such as IL-1 and TNF), facilitating cholesterol transport, promoting immune cell recruitment, and serving as biomarkers for infections, rheumatic disorders, and cardiovascular diseases [27,28]. Total serum cholesterol and triglyceride levels were measured in serum by commercially available enzymatic assays (cholesterol CHOD-PAP 11491458 and triglycerides GPO-PAP 11488872, Roche, Woerden, the Netherlands) according to the manufacturers’ protocols.

### 2.3 Histology and immunohistochemistry

Dissected hearts including the area of the aortic roots were embedded in paraffin after overnight fixation (4% formalin). Serial cross sections (4 μm) of the aortic root were made and a hematoxylin-eosin staining was performed in order to identify the cross-sections containing the AV. Sections of the AV containing the largest surface area of valve tissue were used for (immuno)histochemical analysis depicted below. Serial cross-sections were stained with Elastica von Gieson, Alcian Blue, von Kossa, and Perl's iron for For immunohistochemical analyses, the cross-sections were dewaxed, rehydrated and incubated in methanol/H_2_O_2_ (0.3%) for 30 min to block endogenous peroxidases. Antigen retrieval was performed by the use of citrate buffer, pH 6.0, for 15 min at 100°C using a microwave (CD45 and MAC-3). To detect leukocytes (CD45), the sections were subsequently incubated with rat-anti-mouse CD45 antibody (1:50 dilution, BD Pharmingen, cat. 550539, overnight at 4°C). For the detection of macrophages (MAC-3) the sections were first incubated with normal rabbit serum (1:50 dilution, DAKO, X0902, 10 min, RT) to reduce nonspecific background staining and subsequently with purified rat-anti-mouse MAC-3 (1:30 dilution, BD Pharmingen, clone M3/84, 553322, overnight at RT). Rabbit-anti-rat Immunoglobulines/HRP (1:50 dilution, DAKO, P0450, 30 min at RT) was used as a secondary antibody for both CD45^−^and MAC-3 staining. The sections were subsequently rinsed with PBS and incubated with 3,3′-diaminobenzidine (DAB) for 10 min in the dark. All sections were counterstained with Hematoxylin (Mayer) for 1 min.

### 2.4 Morphometric and immunohistochemical analysis

Histological and immunohistochemical sections were imaged using a PathScan Enabler IV slide scanner (Meyer Instruments Inc., Houston, TX, USA). Digital image analysis was performed for histoanatomical characterization and quantitative assessment. For each mouse, multiple aortic valve sections were analyzed.

Aortic valve thickness was assessed on elastin van Gieson (EVG)-stained sections. For each section, all three valve leaflets were analyzed, and leaflet thickness was measured at the midpoint of the leaflet, corresponding to the thickest region. The mean leaflet thickness per section was calculated by averaging the measurements of the three leaflets. Subsequently, an overall mean valve thickness per mouse was determined by averaging across all analyzed sections.

Elastin content, fibrosis, macrocalcification, and glycosaminoglycans (GAGs) were quantified as the area occupied by each component within the tissue sections using QuickPhoto MICRO 3.0 software. Measurements were normalized to the total valve area and expressed as percentages. For each mouse, mean values were calculated across all analyzed sections. Iron deposition, indicative of intravalvular hemorrhage, was assessed using Perls’ Prussian blue staining and evaluated qualitatively (presence or absence) by light microscopy. Lipid content within the aortic valve (AV) was estimated indirectly by subtracting the areas positive for fibrosis and glycosaminoglycans (GAGs) from the total AV area, with the remaining area considered to represent lipid-rich regions.

Immunohistochemical staining for CD45 and MAC-3 was performed to assess leukocyte and macrophage infiltration in the AV sections. CD45 is a pan-leukocyte marker and therefore identifies multiple leukocyte populations, including lymphocytes, which are known to express high levels of CD45. Quantification of positively stained cells was performed manually using light microscopy. The number of positive cells was normalized to the corresponding AV area and expressed as cells per unit area. Neutrophilic granulocytes were identified morphologically and quantified in a similar manner. The surface area of each AV section was determined using the PathScan Enabler IV slide scanner and QuickPhoto MICRO 3.0 software. For each mouse, the mean number of immune cells per unit area was calculated across all analyzed sections. All analyses were performed by a single investigator in a blinded manner.

### 2.5 Statistics

Statistical analyses were performed using SPSS (Windows version 22.0, IBM Corp., Armonk, NY, USA). Predefined pairwise comparisons were conducted between (i) non-atherosclerotic control mice (C) and atherosclerotic control mice (AC) to assess the effect of the atherosclerotic model, and (ii) atherosclerotic mice without surgery (AC) and with surgery (AS) at each time point (day 5 and day 15) to evaluate the effect of orthopedic surgery.

For each comparison, an independent samples *t*-test was used for normally distributed data, and a Mann–Whitney *U* test for non-normally distributed data. To account for multiple testing, p-values were adjusted using the Benjamini–Hochberg procedure in R (version 3.4.2). Adjusted p-values < 0.05 were considered statistically significant.

Data are presented as mean ± SD. Graphs were generated using GraphPad Prism (version 7.0).

## 3 Results

### 3.1 SAA levels and serum lipids: model validation

As depicted above, AVs were isolated from a previously performed animal experiment. In these mice, surgery induced a significant increase in SAA levels, indicative for induced systemic inflammation [18]. Surgery also temporally, but significantly, lowered serum triglyceride levels at day 1 post-surgery that returned to baseline levels at day 5 post-surgery [18].

### 3.2 Valve thickness, fibrosis and calcification

**Fig. 1:**
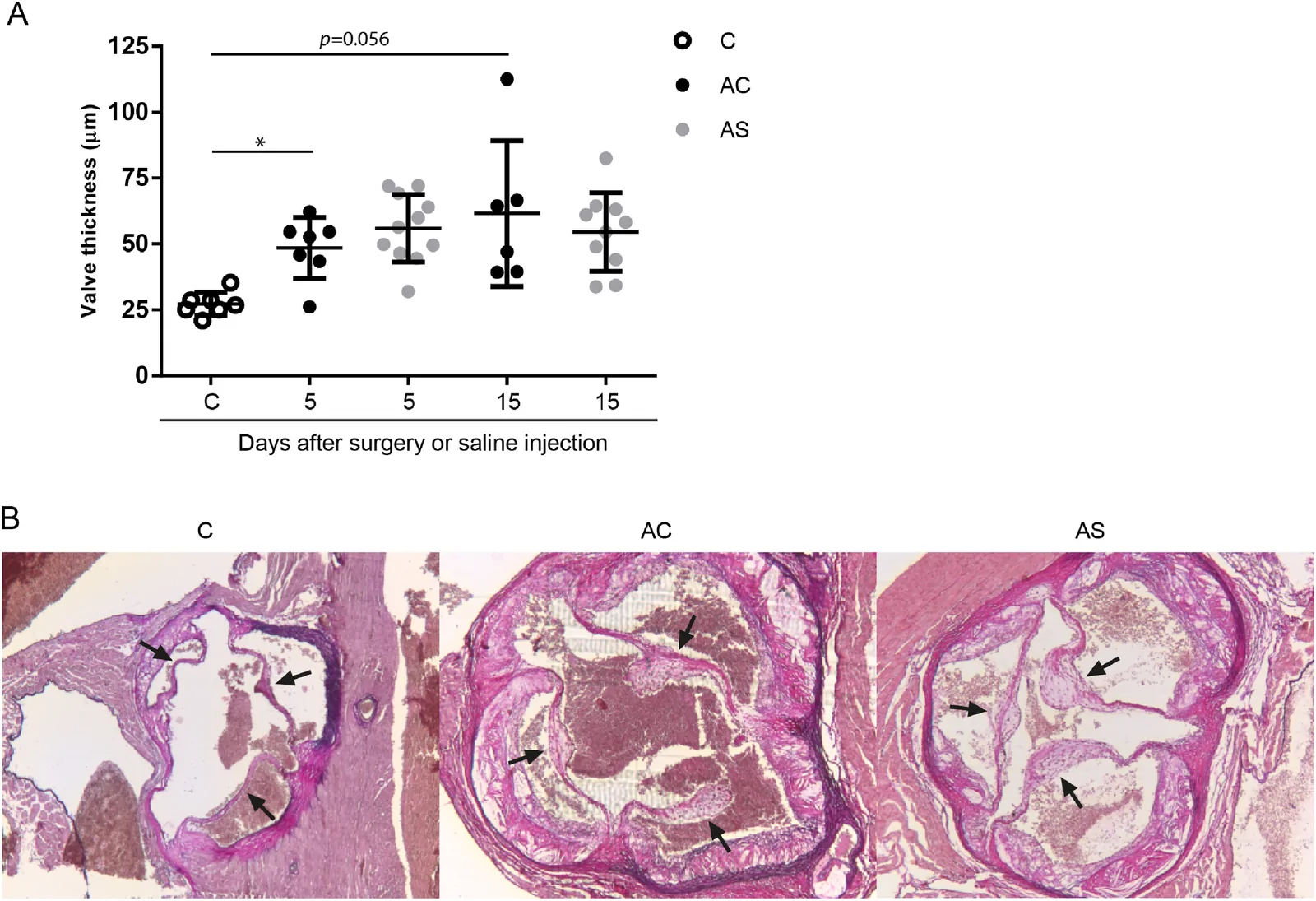
Effect of orthopedic surgery on aortic valve thickness. A: Valve thickness (μm) is displayed per group. *ApoE*^*−/−*^ mice with surgery (AS) or without surgery (AC) were sacrificed at day 5 or 15 post surgery/post saline injection. C indicates *C5*7BL*/6* control mice. Values are mean ± SD. *p < 0.05, **p < 0.01. B: Representative EvG cross sections of the AV from *C5*7BL*/6* mice and *ApoE*^*−/−*^ mice (AC: atherosclerotic control, no surgery; AS: with surgery). Magnification was set on 50x for all groups. Arrows indicate aortic valve leaflets.

Three groups were studied: ApoE−/− mice that underwent the surgical procedure (AS group), ApoE −/− mice without surgery (AC group) and non-atherosclerotic mice C57BL/6 (C group). In the AC group, AV thickness was significantly larger on day 5 (48.5 μm [37.8-59.3]; *p = *0.004) and non significantly increased on 15 day (61.58 μm [32.6-90.6]; *p = *0.056) compared to the C group (27.3 μm [23.2-31.4]; Fig. 1A), supporting atherosclerotic AV changes in ApoE−/− mice. Surgery, however, did not further increase AV thickness on both 5- and 15 days. (Fig. 1A).

**Fig. 2:**
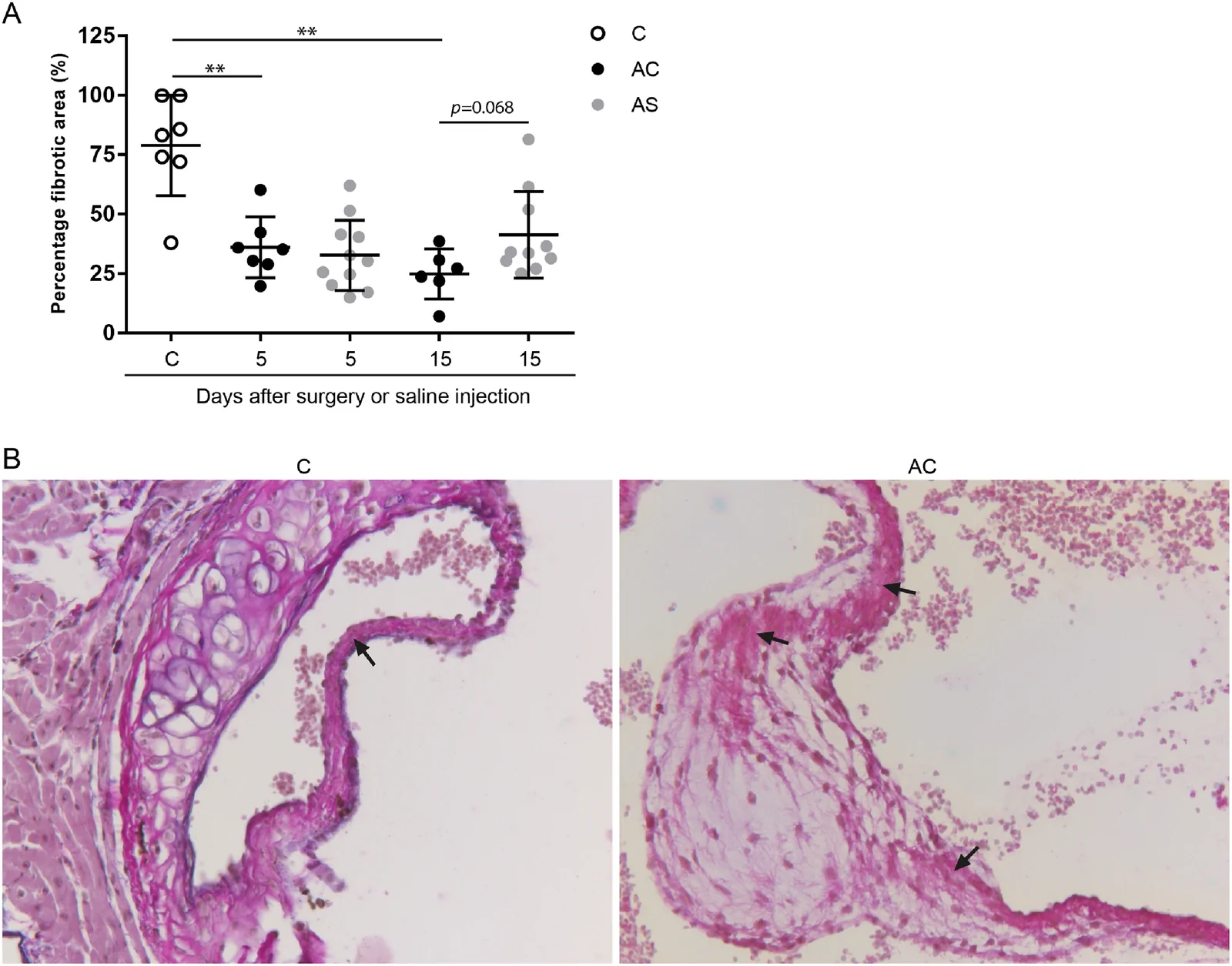
Effect of orthopedic surgery on the percentage of fibrosis in the aortic valve. A: Percentage of fibrosis (%) based on EvG staining in the AV of *C5*7BL*/6* control mice (C) and *ApoE*^*−/−*^ mice with surgery (AS) and without surgery (AC) sacrificed at day 5 or 15 post surgery/post saline injection. Values are mean ± SD. *p < 0.05, **p < 0.01. B: Representative EvG staining in AV leaflets. Arrows indicate fibrotic area located in the AV. Magnification 200x.

It is known that the extracellular matrix (ECM) of the valve contributes to disease progression by promoting fibrosis and calcification [29]. In the AC group the area of fibrotic tissue was significantly decreased both on day 5 and 15 to respectively 36.1% [24.3-47.8](*p = *0.002) and 24.9% [13.8-35.9](*p = *0.001) compared to the C group (78.9% [59.4-98.5]). Surgery did not affect the fibrotic area on day 5. Although the fibrotic area was higher in the surgery group at day 15 (41.3% [28.3–52.2] vs. 24.9% [13.8–35.9]), this difference did not reach statistical significance (p = 0.068; Fig. 2A).It has to be noticed that AV leaflets in the C group were almost homogenous fibrotic, whereas in the AC and AS group the edges of the valves were homogenous fibrotic while in the thickened centers of the valves a more diffuse fibrosis pattern was found (Fig. 2B). No difference was observed between the AC and AS group in the distribution of the fibrotic tissue in the AV leaflets (not shown).

To detect putative calcium deposits a von Kossa staining was performed. However, no calcium deposits were found in the AV in none of the different mice groups (data not shown).

### 3.3 Glycosaminoglycans (GAGs), lipids and intraleaflet hemorrhage

**Fig. 3:**
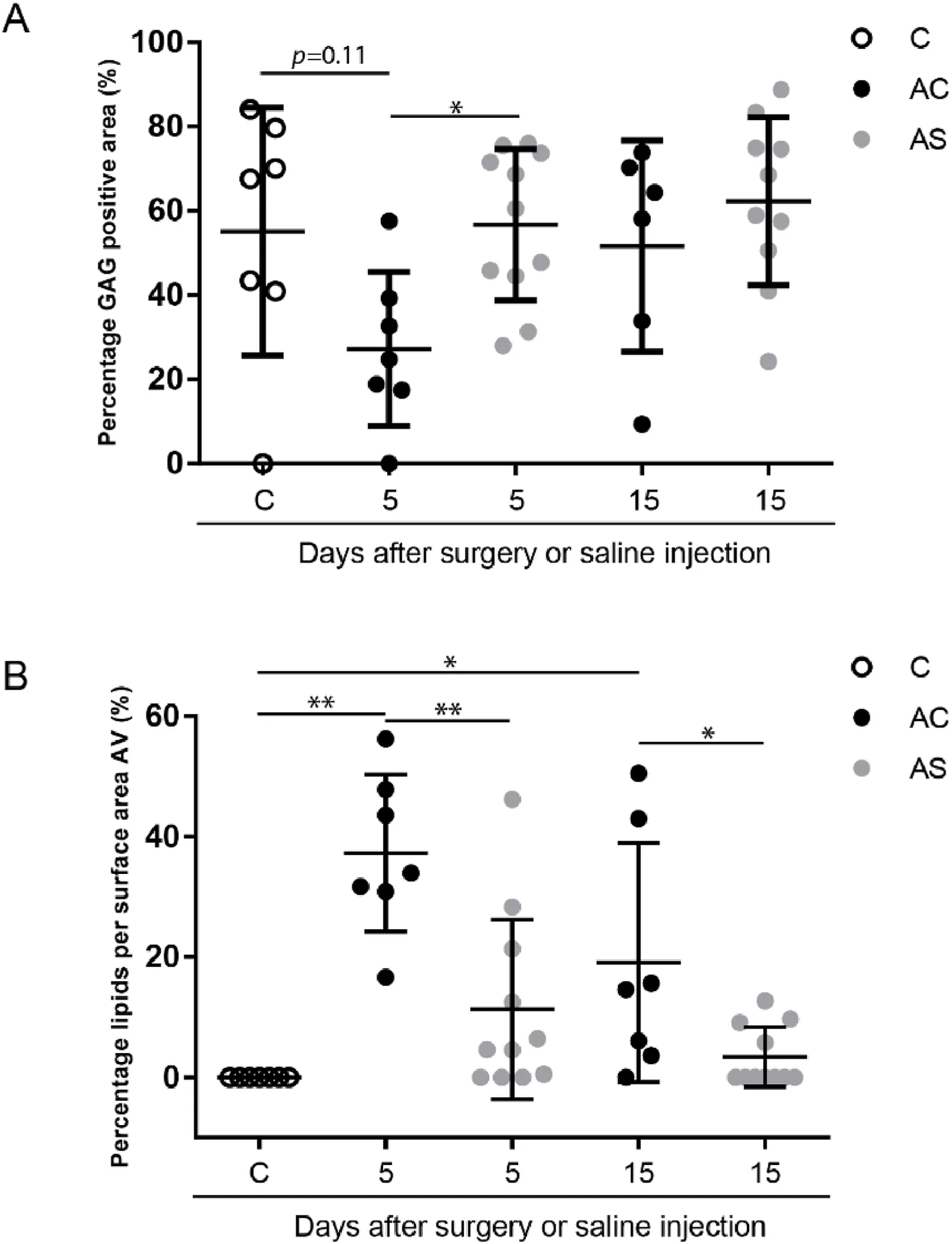
Effect of orthopedic surgery on glycosaminoglycans (GAGs) and estimated lipid-rich area in the aortic valve. A: Percentage of GAGs (%) based on Alcian Blue staining in the AV of *C5*7BL*/6* control mice (C) and *ApoE*^*−/−*^ mice with surgery (AS) and without surgery (AC) sacrificed at day 5 or 15 post surgery/post saline injection. Values are mean ± SD. *p < 0.05, **p < 0.01. B: Percentage of estimated lipid-rich area (%) in the AV of *C5*7BL*/6* control mice (C) and *ApoE*^*−/−*^ mice with surgery (AS) and without surgery (AC) sacrificed at day 5 or 15 post surgery/post saline injection. The estimated lipid-rich area in the AV was measured by subtracting the fibrotic area and GAG positive area in the AV from the total surface area AV. This outcome times 100 percent indicates the percentage of estimated lipid-rich area in the AV. Values are mean ± SD. *p < 0.05, **p < 0.01.

Glycosaminoglycans (GAGs) form an important component of the ECM involved in AS. We analyzed GAGs in the AV using Alcian Blue staining. No significant differences were observed in the GAG positive area of the AV between AC and C groups at both 5- and 15 days (Fig. 3A). Surgery caused a significant increase in the area of GAGs on day 5 (56.7% [44.7-68.7] in the AS group) compared to the AC group (27.3% [10.3-44.2]; *p = *0.016), but not at 15 days post-surgery (Fig. 3A).

We further observed a significant increase in the estimated lipid-richt area in the AC group on both day 5 (37.3% [25.2-49.3]; *p = *0.0011) and day 15 (19.1% [0.7-37.4]; *p = *0.044) compared to the C group (0%; Fig. 3B). Remarkably, after surgery the estimated lipid-rich area significantly decreased on day 5 (11.3% [1.3-21.3] vs. 37.3% [25.2-49.3]; *p = *0.008) compared to AC mice. At 15 days also a significant decrease was observed (3.4% [0.06-6.7] vs. 19.1% [0.7-37.4], *p = *0.031; Fig. 3B).

Next, we analyzed putative hemorrhage of the AV. We did not find iron deposits, indicative for old hemorrhages in the valves, in any of the groups.

### 3.4 Inflammatory cells

**Fig. 4:**
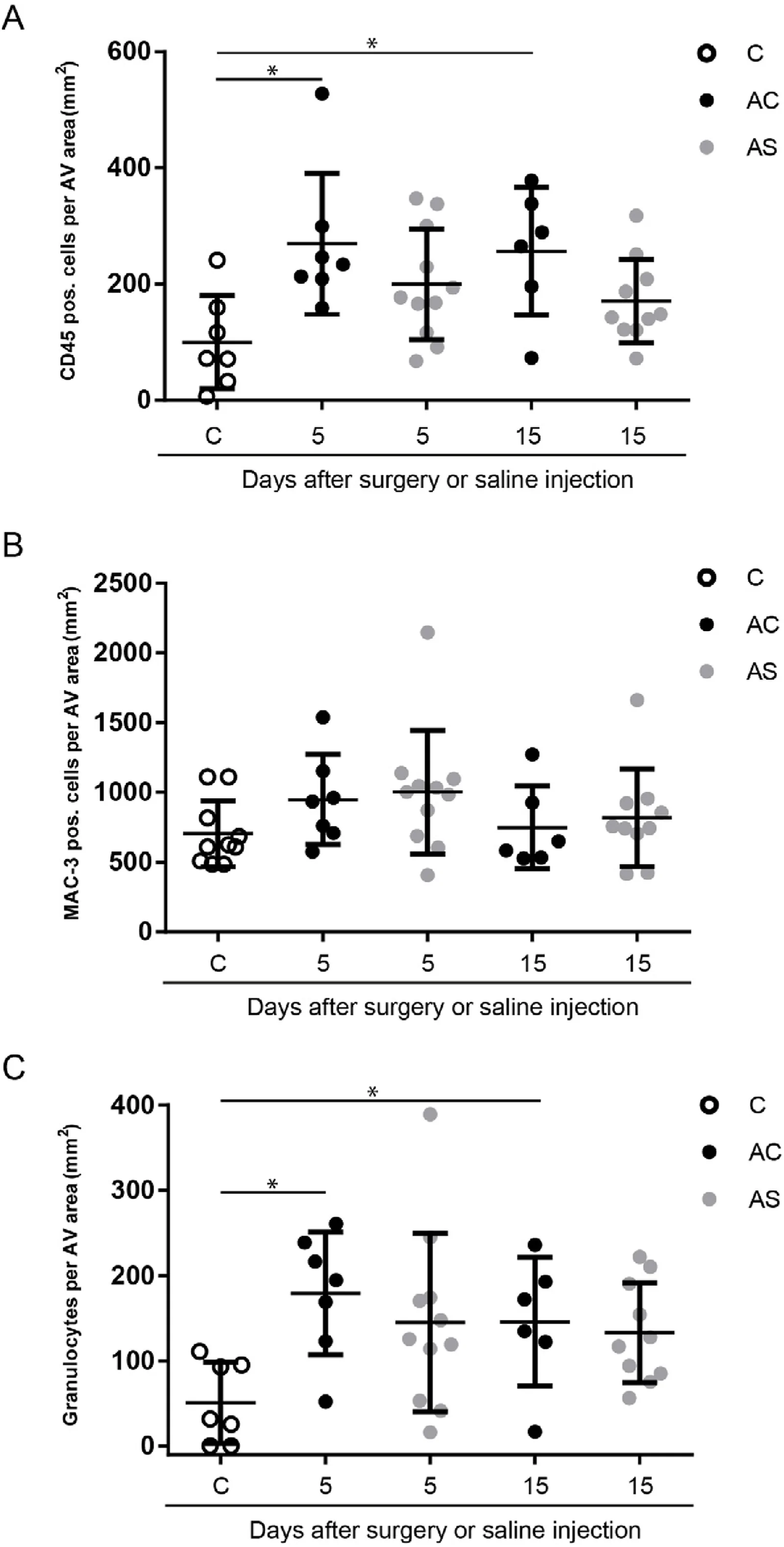
Effect of orthopedic surgery on inflammatory cells in the aortic valve. A: CD45 positive leucocytes in the AV divided by total surface area of the AV (cells/mm^2^). B: MAC-3 positive macrophages in the AV divided by total surface area of the AV (cells/mm^2^). C: Neutrophilic granulocytes in the AV divided by total surface area of the AV (cells/mm^2^). *ApoE*^*−/−*^ mice with surgery (AS) or without surgery (AC) were sacrificed at day 5 or 15 post surgery/post saline injection. C indicates *C5*7BL*/6* control mice. Values are mean ± SD. *p < 0.05, **p < 0.01.

We finally analyzed the effect of surgery on the number of intra-valvular inflammatory cells. In the AC group a significant increase in the number of CD45 positive leukocytes was found both on day 5 (*p = *0.026) and day 15 (*p = *0.026) compared with the C group (Fig. 4A). Surgery did not have a significant effect hereon (Fig. 4A). No significant difference in the number of intra-valvular MAC-3 positive macrophages was found between the groups (Fig. 4B). In the AC group we however found a significant increase in the number of neutrophilic granulocytes on day 5 (*p = *0.008) and day 15 (*p = *0.038) compared to the C group, which was not further enhanced at 15 days (Fig. 4C). Surgery didn't have an effect hereon (Fig. 4C).

## 4 Discussion

We hypothesized that systemic inflammation induced by orthopedic surgery would exacerbate AV disease. We found that atherosclerosis itself significantly increased AV thickness, accompanied by a significant decrease in the relative fibrotic area, an increase in the estimated lipid-rich area, and increased numbers of CD45-positive leukocytes and neutrophilic granulocytes. Comparison between atherosclerotic mice with or without surgery revealed that orthopedic surgery significantly increased GAG content in the AV at day 5 post-surgery. A higher fibrotic area was observed at day 15, although this difference did not reach statistical significance. Surgery also significantly decreased the estimated lipid-rich area on both day 5 and day 15. Finally, surgery did not affect AV thickness or inflammatory cell infiltration at the investigated time points. These findings suggest that orthopedic surgery is associated with compositional remodeling of the AV without affecting valve thickness or inflammatory cell infiltration at the investigated time points.

The molecular mechanisms underlying the observed valve remodeling remain unknown. One possible explanation is that tissue injury caused by orthopedic surgery leads to the release of damage-associated molecular patterns (DAMPs), which may activate innate immune pathways, including Toll-like receptor (TLR) signaling, thereby inducing a systemic inflammatory response [[30], [31], [32], [33]]. Previous experimental studies have implicated TLR signaling in both the pathogenesis of atherosclerotic plaque formation and calcific aortic valve disease [34,35]. Taken together, these observations raise the possibility that surgery-induced systemic inflammatory signaling contributes to the compositional changes observed in the aortic valve. However, the present study did not investigate circulating cytokines, TLR or complement activation, or valve interstitial cell (VIC) activation. Consequently, the proposed mechanism remains hypothetical and warrants further investigation.

Whether the observed remodeling results in impaired AV function remains unknown, as no functional measurements, such as echocardiographic or hemodynamic assessments, were performed in the present study. Nevertheless, previous studies have shown that alterations in fibrosis and/or GAG content affect valve functionality, including increased leaflet stiffness in aging human valves [36,37]. Furthermore, major non-cardiac surgery, including orthopedic surgery, has previously been associated with accelerated hemodynamic progression of AS in humans, although the direct effects on valve morphology were not investigated [23].

The absence of increased inflammatory cell infiltration in the AV at day 5 and day 15 suggests that the observed extracellular matrix remodeling is unlikely to be explained by sustained local accumulation of inflammatory cells. However, this should not be interpreted as evidence that inflammatory cells are not involved in the initial response to orthopedic surgery. Our previous study demonstrated that systemic inflammation peaks within the first postoperative day, as reflected by a marked increase in serum amyloid A (SAA), accompanied by increased inflammatory cell infiltration in the atherosclerotic plaque [18]. Because AV tissue was only available at day 5 and day 15, transient inflammatory cell infiltration during the early postoperative phase cannot be excluded. It is therefore possible that inflammatory cells were recruited shortly after surgery and had already resolved before the investigated time points. Future studies including earlier postoperative time points are required to further elucidate the temporal relationship between systemic inflammation and AV remodeling.

The significant increase of the inflammatory content of especially CD45 positive leukocytes and neutrophilic granulocytes in atherosclerotic changed valves coincided with a decrease in valve fibrosis. However, as stated above, we cannot exclude that the observed relative decrease in fibrosis is exclusively caused by the increase in valve thickness – coinciding with increased AV area – and not by a decrease of fibrotic tissue itself.

Since no difference in AV thickness was observed between atherosclerotic control and surgery-subjected mice, the numerically higher fibrotic area observed on day 15 may reflect remodeling processes. However, this difference did not reach statistical significance and should therefore be interpreted with caution. Previous studies already revealed a link between fibrosis and acute inflammation: it was shown that acute phase proteins, such as serum amyloid P, are important inducing signals of fibrosis [38,39]. Furthermore, we observed an increase of GAGs in the atherosclerotic valves upon surgery and it is known that GAGs recruit inflammatory cells [40,41]. We, however, did not find an increase of inflammatory cells in the AV upon surgery.

A remarkable difference with our previous study is that we found that surgery caused a significant increase of atherosclerotic plaque area of the aorta, which was mainly due to necrotic core enlargement. In contrast, we did not find increased AV thickness, while the estimated lipid-rich area was decreased. The mechanisms underlying these differences remain unclear. Indeed, previous studies have shown significant differences between the atherosclerotic plaques in arteries and stenotic valves in terms of response to lipid lowering therapies and lipid content. While statins are effective in preventing ischemic atherosclerotic events, they are ineffective in delaying the progression of aortic stenosis [42]. At the tissue level, stenotic valves, unlike atherosclerotic plaques, do not contain areas of extensive lipid-associated necrosis. Moreover, the location of (oxidized) lipid deposition in atherosclerotic plaques of arteries is the core region, whereas in the atherosclerotic changed valves lipids accumulate in the sub endothelial layer [43]. Furthermore, the temporal changes in circulating serum lipids did not correspond to the estimated lipid-rich area observed locally in the AV. Surgery induced a transient decrease in serum triglyceride levels on day 1 post-surgery, which returned to baseline by day 5 [18], whereas the estimated lipid-rich area in the AV remained reduced at day 5. This indicates that the lipid metabolism in AS is hard to comprehend. An important limitation of the present study is that lipid accumulation was not measured directly. Instead, the estimated lipid-rich area was derived indirectly by subtracting the fibrotic and glycosaminoglycan-positive areas from the total valve area. Although this approach provides an estimate of valve composition, it cannot distinguish lipid deposition from other unstained tissue components. Therefore, these findings should be interpreted with caution. In sum, it can be stated that surgery had a similar effect in arterial atherosclerosis and AS considering the effect on the number of inflammatory cells, at least at the time points studied, whereas the morphological changes were different.

No calcification or intra-valvular hemorrhage was observed in any of the experimental groups, indicating that the present ApoE−/− model represents an early stage of aortic valve disease rather than advanced calcific aortic valve disease. Although we cannot completely exclude a role for calcification, previous studies using a similar ApoE−/− model likewise failed to detect valve mineralization by von Kossa staining despite evidence of ongoing osteogenic activity within the valve [44]. Therefore, our findings should be interpreted in the context of early valve remodeling, and caution is warranted when extrapolating these results to the progression of human calcific aortic valve disease.

An important limitation of the present study is that the original animal experiment was designed to investigate the effects of orthopedic surgery on atherosclerotic plaque characteristics rather than aortic valve remodeling. Consequently, the study was not specifically powered for aortic valve endpoints, and the relatively small group sizes after subdivision by time point may have limited the ability to detect more subtle differences between groups. The present findings should therefore be regarded as exploratory and hypothesis-generating and warrant confirmation in dedicated experimental studies of aortic valve pathology and, ultimately, in human studies.

In conclusion, orthopedic surgery was associated with compositional remodeling of the AV. These findings suggest that systemic inflammatory responses may contribute to early AV remodeling, although the underlying molecular mechanisms were not investigated in the present study. As the present ApoE−/− model represents early-stage aortic valve disease without overt valve calcification, caution is warranted when extrapolating these findings to the progression of human calcific aortic valve disease. Future studies are needed to determine the molecular pathways involved and to establish whether these compositional changes affect valve functionality.

## Author contributions

AvB: performed the investigation, conducted formal analysis, visualized the data, wrote the original draft, and applied revisions; WWF: proposed the research idea, contributed to the investigation, and revised the manuscript; IPAZ: contributed to the investigation and revised the manuscript; WNvW: conducted formal analysis and revised the manuscript; YMS: contributed to the study design and methodology, provided supervision, and revised the manuscript; ABAV: acquired funding, provided supervision, and revised the manuscript; PAJK: proposed the research idea, contributed to the study design and methodology, provided supervision, and revised the manuscript; HWMN: proposed the research idea, contributed to the study design and methodology, acquired funding and resources, provided supervision, and revised the manuscript.

## Data availability statement

The data that support the findings of this study are available from the corresponding author upon reasonable request after publication.

## Funding

This work was financially supported by the 10.13039/100019741Amsterdam Cardiovascular Sciences, the Netherlands and 10.13039/100006520Edwards Lifesciences.

## Conflict of interest

The authors declare that they have no known competing financial interests or personal relationships that could have appeared to influence the work reported in this paper.
